# Induction of Heat Shock Proteins in the Therapy of Type 2 Diabetes and Metabolic Syndrome

**DOI:** 10.1016/j.ebiom.2014.11.006

**Published:** 2014-11-07

**Authors:** Philip L. Hooper

**Affiliations:** Division of Endocrinology, Metabolism, and Diabetes, University of Colorado Anschutz Medical Campus, Aurora, CO, United States

Exercise is one of the best therapies, if not the best, for the prevention and treatment of type 2 diabetes. Kondo, Araki, Kai and colleagues, in this issue of EBioMedicine, introduce a device that mimics exercise (1). When applied to subjects with metabolic syndrome (MS) or type 2 diabetes (t2DM) the therapy improves the diverse parameters inherit to metabolic syndrome (MS) and type 2 diabetes (t2DM)—inflammation, hypertension, glycemia, dyslipidemia, abdominal obesity, body weight, and insulin signaling. The results are a quantum leap toward understanding type 2 diabetes and treating it. The researchers are to be congratulated in their single-minded quest to move from the bench to the bedside. As they sought methods to duplicate the beneficial effects of exercise, their research moved from heat, to heat shock proteins (Hsps, also known as stress proteins), to a tailored electrical-heat stimulation that maximizes Hsp induction.

Specifically, Kondo and colleagues studied 40 patients with MS or t2DM in a randomized crossover trial of 12 weeks of therapy with mild electric stimulation and heat shock (MES + HS) versus no intervention for 12 weeks. The device transmits direct electric current and heat through the upper abdomen, fitting like a cumberbund. MES + HS was applied for 1 h four times per week. Improvements in glycemic, lipid, and fat deposition measurements were more pronounced in t2DM than MS. Of particular note was a .72% reduction in A1c in t2DM subjects with higher baseline levels (7.6–10.0%) (Kondo et al., 2014). This level of reduction is similar to that seen by recently introduced drugs like sitagliptin (a dipeptidyl peptidase 4 (DPP-4) inhibitor) that drops A1c .6% when used in monotherapy (Zerilli and Pyon, 2007).

A remarkable 55% drop in albuminuria was observed in t2DM with a drop in serum creatinine. The fall in albuminuria is in the same order of magnitude as observed with ACE inhibitor therapy (Yilmaz et al., 2010). The same research group has demonstrated MES + HS reduced renal function loss in an animal model of renal failure (Koga et al., 2012).

Regarding its efficacy in reducing inflammation in MS, MES + HS dropped hs-CRP by 54%. hs-CRP in t2DM improves prediction of cardiovascular disease and has been suggested that its measurement be added to the definition of metabolic syndrome. The reduction in inflammation cannot be understated, as cardiovascular disease is indeed the major cause of mortality in these disease states (Haffner, 2006). The 54% drop in hs-CRP compares favorably with the drop observed with atorvastatin (35%) in t2DM patients (Sindhu et al., 2011).

Low intracellular Hsps in insulin sensitive tissues like skeletal muscle and liver may be the near sentinel event in the development of MET or t2DM. Low muscle intracellular Hsp precedes abnormal metabolic function by four years in non-human primates which is comparable to a decade in humans (Chichester et al., 2014). Furthermore, normo-glycemic identical twins of siblings with impaired glucose tolerance have low muscle Hsp expression. Restoring the low Hsps via exercise or bioactive Hsp inducers normalizes the pathologic features of MS and t2DM. Hsp induction stimulates survival pathways like AMPK that limits fat storage while activating fat catabolism. Mitochondria biogenesis and mitochondrial function are improved by Hsp induction—augmenting reduction in adipose stores. Inflammation is markedly improved with major reductions in CRP and cytokine activation—blocking the vicious cycle of inflammation, reduced insulin signaling which further impairs Hsp expression and thus promotes more inflammation (Hooper et al., 2014).

MS and t2DM put organs in peril—from neuropathies, to retinopathies, to myopathies, to nephropathies, to hepatic seatosis, to dementia. Restoring Hsps in diabetic animal models limits the diabetic complications. Reduction in systemic inflammation will likely reduce the vascular disease so prevalent in these diseases. Mild electric stimulation (MES) indeed reduces nephropathy and fatty liver. Similarly, MES + HS augments pancreatic beta-cell function in animal diabetic models (Kondo et al., 2012).

The health care provider is often frustrated by limited efficacious therapies available to prevent and treat the metabolic disease epidemic. While life-style modification remains the initial and most effective therapy, long-term compliance often does not endure. Can the MES + HS device be used throughout the course of this syndrome-complex? Certainly, a diabetic individual with proteinuria and an elevated serum creatinine is a candidate for a MES + HS device to use in their home. The same is true for hepatic seatosis with progressive liver injury. Of course, how will the device be used in clinics or at home? How expensive will it be? Can a similar device be used for other therapies—arthritis, wound healing, and heart failure—where an augmented cellular stress response may be beneficial? MES + HS efficacy is comparable if not better than present mainstay pharmaceutical agents. Furthermore, MES + HS is broadly effective in normalizing the whole spectrum of pathologic defects associated with MS and t2DM—not unlike the effect of regular exercise.

## Disclosure

The author declared no conflicts of interest.
